# Simultaneous multi-slice MR imaging of the hip at 3 T to reduce acquisition times and maintain image quality

**DOI:** 10.1186/s12891-018-2342-x

**Published:** 2018-12-13

**Authors:** Mayuko Haraikawa, Masashi Suzuki, Kaiji Inoue, Eito Kozawa, Junji Tanaka, Mamoru Niitsu

**Affiliations:** Department of Radiology, Saitama Medical University, 38 Morohongo, Moroyama-machi, Iruma-gun, Saitama, 350-0495 Japan

**Keywords:** Simultaneous multi-slice, Turbo spin echo, Magnetic resonance imaging, Hip joint, Fast imaging, Musculoskeletal disorders

## Abstract

**Background:**

Newly developed simultaneous multi-slice (SMS) scans are now being introduced as a clinical application in neuroimaging. We examined the feasibility of SMS scans for joint imaging. The purpose of the present study was to prospectively compare the resolution and specific absorption rate (SAR) obtained using SMS to those of conventional methods in hip joint magnetic resonance imaging (MRI) and establish whether imaging times may be reduced using SMS in 3 T MRI and if image quality is affected.

**Methods:**

Twenty-one patients (4 men and 17 women, average age, 51.5 years, range: 22 to 76 years) with hip pain underwent MR examinations of the unilateral hip joint. Three board-certified radiologists independently and blindly evaluated the images obtained with and without SMS using window and level settings and magnification according to personal preferences. Individual SAR values were measured for each protocol. A Wilcoxon signed-rank test and a *t*-statistic test were used for statistical analyses. Signal-to-noise ratio (SNR) was also compared using a phantom.

**Results:**

SMS imaging maintained equivalent image quality to conventional imaging for evaluating the morphology of the hip joint, and also reduced imaging times by approximately 40%. SMS images had significantly higher SAR values than conventional images. The rate of difference (SMS/conventional) in SNR ranged between 80 and 111%.

**Conclusions:**

Based on its significantly lower acquisition times and the maintenance of similar image quality to conventional imaging, SMS may be applied to morphological evaluations of hip joint disorders without significantly increasing SAR.

## Background

Magnetic resonance imaging (MRI) with high tissue contrast is essential for diagnostic imaging of the hip joint [1]. In order to facilitate initial treatments, cartilage, labrum, ligament, and tendon diseases need to be precisely diagnosed in the early stages using MRI [2]. MRI plays an important role in decision making for surgical treatments, the alleviation of symptoms, and improved prognoses [3, 4].

MRI with a high magnetic field strength and phased array coil provides a higher signal-to-noise-ratio (SNR) and enables thin-sliced images to be obtained while maintaining high spatial resolution. In 2D multi-slice imaging, each slice excitation and signal reception are conducted sequentially, with the shortest repetition time (TR) becoming longer with increases in the number of slices. Therefore, imaging times are prolonged. Prolonged scanning duration may inconvenience patients and result in unwarranted movements, leading to blurred images due to motion artifact. Furthermore, patients with hip joint pain move more. Decreases in image acquisition times improved patient satisfaction and cost effectiveness by reducing scan times.

Simultaneous multi-slice (SMS) imaging is a method that reduces the scan time of 2D imaging by simultaneously exciting several slices [5, 6]. Since the information obtained on simultaneously excited slices is superimposed in the MR signal received, it is divided into each slice with slice-GRAPPA (Generalized Auto Calibrating Partially Parallel Acquisitions) reconstruction. SMS is now mainly applied to echo planar imaging sequences, and has been used for diffusion and functional MRI [7–9]. If SMS becomes applicable to turbo spin echo (TSE) sequences, which are commonly used, it will be more useful [10]. The scan parameters that need to be changed in order to reduce the scan time in SMS include TR, the turbo factor, and concatenations. These parameters are related to image contrast. Moreover, care is needed in order to avoid exceeding the regulatory limits of the specific absorption rate (SAR).

The objective of the present study was to prospectively compare the resolution and SAR obtained using SMS to those of conventional methods in hip joint MRI and establish whether imaging times may be reduced using SMS in 3 T MRI and if image quality is affected.

## Methods

### Patients

**Fig. 1:**
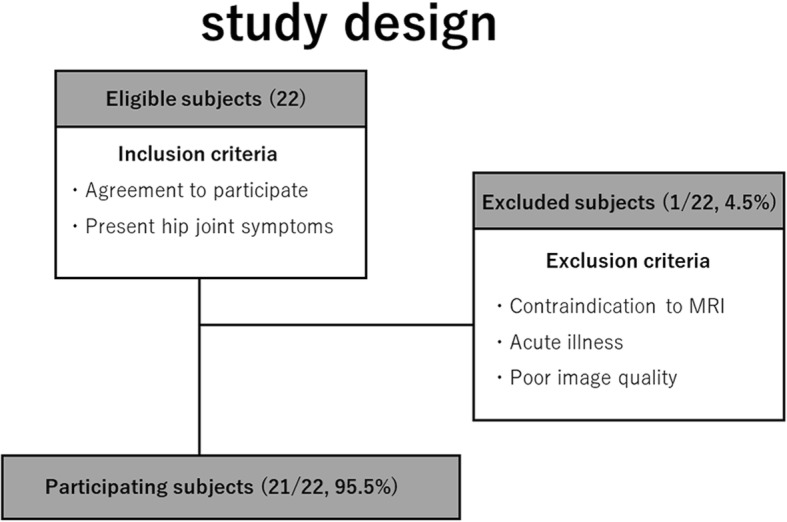
Study design

The study design is outlined in Fig. 1. Twenty-one patients (4 men and 17 women, average age: 51.5 years, age range: 22 to 76 years) with hip pain and a presumed acetabular labral tear underwent MR examinations of the unilateral hip joint. All patients presented at the hip surgery clinic of the orthopedic department of our institution. This prospective study was performed between November 2016 and March 2017, was approved by the local Ethics Committee, and written informed consent was obtained from all patients prior to MR examinations.

### MRI

All examinations were performed on a 3.0 T whole-body scanner (MAGNETOM Skyra; Siemens Healthcare GmbH, Erlangen, Germany) and a 18-channel body array coil. In addition to our routine protocols using TSE sequences, including oblique coronal proton-density weighted (PDW), oblique axial T1-weighted (T1W), and oblique coronal fat-saturated T2-weighted (FS-T2W) imaging, SMS protocols using a prototype sequence were also acquired in an identical geometry. All SMS images were acquired in the latter half of examinations.

Fixed sequence parameters in all protocols were as follows: number of slices: 30; slice thickness: 2.5 mm; slice gap: 0.25 mm; field-of-view (FOV): 160 × 160 mm^2^. Other individual parameters of conventional and SMS imaging are shown in Table 1.

**Table 1:** Scan parameters of conventional and SMS scans

	Conventional			SMS		
	PDW	T1W	FS-T2W	PDW	T1W	FS-T2W
AT	4:36	3:42	3:58	2:22	2:17	2:56
Total AT	12:16			7:35		
TR (ms)	2000	712	3500	2000	650	3500
TE (ms)	20	27	61	21	14	62
Averages	1	2	2	1	1	2
Concatenations	2	3	2	1	2	1
Flip angle (deg)	150	120	150	150	120	150
Fat suppression	none	none	CHESS	none	none	CHESS
Base resolution	320	384	320	320	384	320
Phase resolution (%)	100	75	70	100	75	70
Parallel imaging	none	GRAPPA	GRAPPA	CAIPIRINHA	CAIPIRINHA	CAIPIRINHA
Acceleration factor phase	–	2	2	1	1	1
Acceleration factor slice	–	–	–	2	2	2
FOV shift factor	–	–	–	2	2	2
Bandwidth (Hz/Px)	240	250	220	240	250	279

*PDW* proton-density weighted, *T1W* T1-weighted, *FS-T2W* fat-saturated T2-weighted, *SMS* simultaneous multi-slice, *AT* acquisition time, *TR* repetition-time, *TE* echo time, *GRAPPA* generalized auto calibrating partially parallel acquisitions, *CAIPIRINHA* controlled aliasing in parallel imaging results in higher acceleration, *CHESS* chemical shift selective, *FOV* field-of-view

### Image evaluation and analysis

**Fig. 2:**
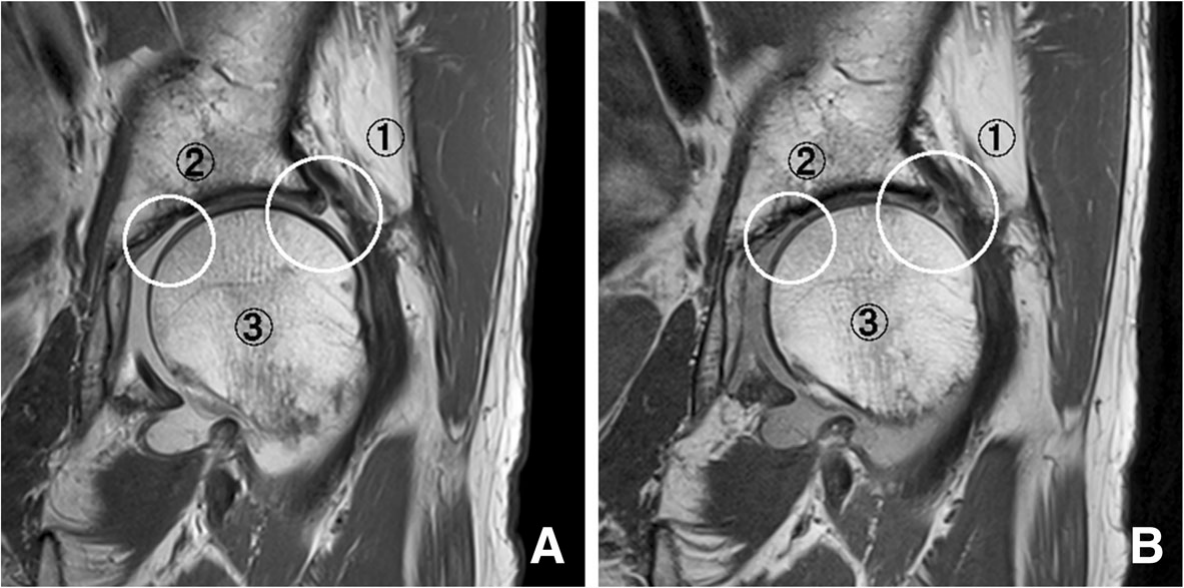
A 76-year-old woman with left hip pain. Oblique coronal proton density-weighted images with conventional (**a**) and simultaneous multi-slice (SMS) sequences (**b**). Circles indicate areas for image evaluations: ➀ acetabular labrum, ➁ articular cartilage, ➂ bony trabecula. Joint fluid in the conventional image appears brighter than that in SMS in this patient

**Fig. 3:**
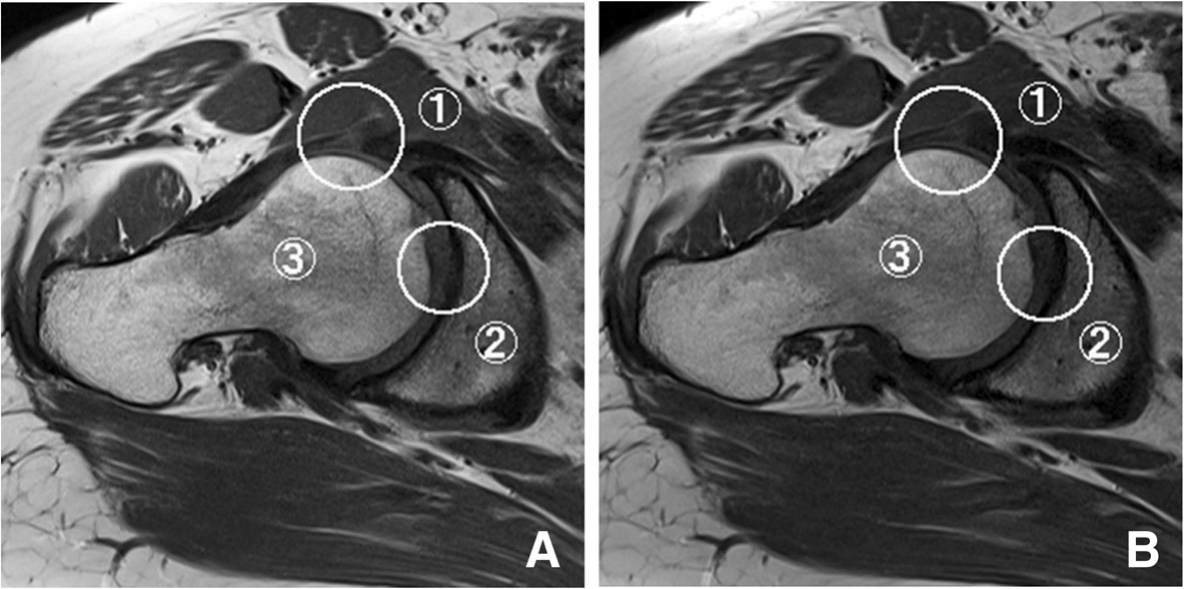
A 50-year-old woman with right hip pain. Oblique axial T1-weighted images with conventional (**a**) and simultaneous multi-slice sequences (**b**). Circles indicate areas for image evaluations: ➀ acetabular labrum, ➁ articular cartilage, ➂ bony trabecula

**Fig. 4:**
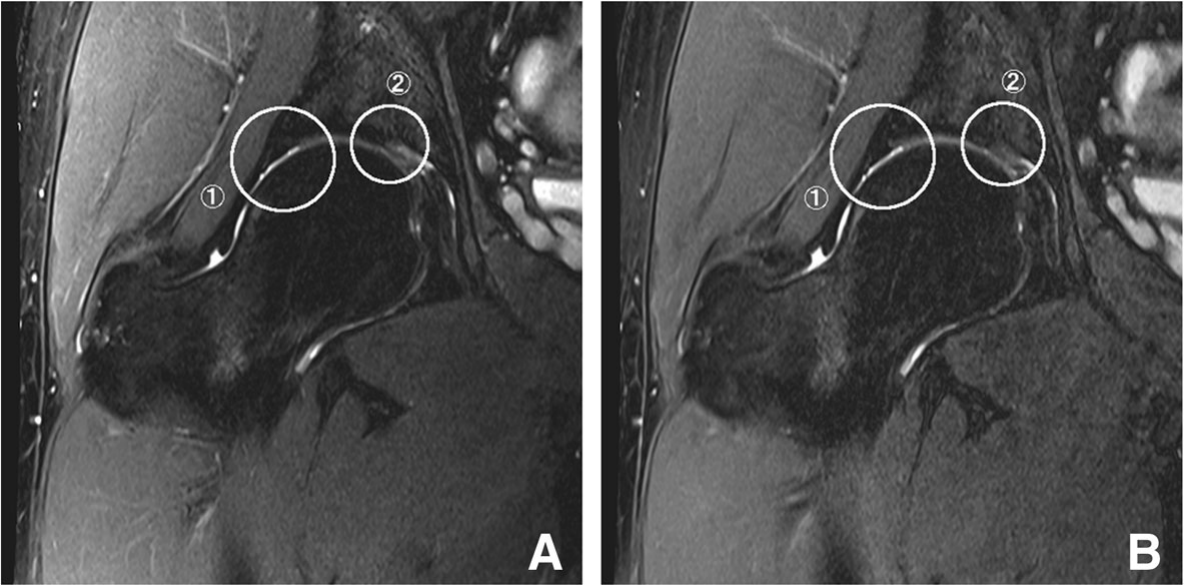
A 22-year-old man with right hip pain. Oblique coronal fat-saturated T2-weighted images with conventional (**a**) and simultaneous multi-slice sequences (**b**). Circles indicate areas for image evaluations: ➀ acetabular labrum, ➁ articular cartilage

We initially excluded some MR examinations with poor image quality from the analysis. Three board-certified radiologists (with 31, 26, and 19 years of experience in musculoskeletal MRI) evaluated pairs of images with and without SMS. Delineative imaging quality was assessed in the delineation of the following structures: the acetabular labrum (labrum), articular cartilage (cartilage), and bony trabecula of the femoral neck (trabecula) (Figs. 2, 3 and 4). The evaluation incorporated the recognition of normal structures as well as pathologies, including labral tears, cartilage defects, and trabecular irregularities. The evaluation also included image blurring, edge sharpness, contrast, and flow/motion artifacts. The labrum and cartilage were evaluated on PDW, T1W, and FS-T2W, while the trabecula was assessed on PDW and T1W. The reviewers independently and blindly evaluated images using window and level settings and magnification according to personal preferences.

Delineative quality was rated using a five-point scale: Conventional (CNV) + 2: the conventional image is superior to SMS, CNV + 1: the conventional image is better than SMS, 0: the conventional image is equal (EQUAL) to SMS, SMS + 1: SMS is better than the conventional image, SMS + 2: SMS is superior to the conventional image.

The five-point evaluation scale was scored as follows: + 2 (CNV + 2), + 1 (CNV + 1), 0 (EQUAL), − 1 (SMS + 1), and − 2 (SMS + 2). A score for each sequence represented the difference between the two groups being compared. A Wilcoxon signed-rank test was used for the statistical analysis. Data analyses were performed with the software JMP® 12 (SAS Institute Inc., Cary, NC, USA), and *p* < 0.05 was considered to be significant.

### SAR

Individual SAR values predicted by the scanner system before the scan (0–100%) were recorded. The average and standard deviation (SD) were calculated in every sequence for all patients. Values with and without SMS were compared through the *t*-statistic test. A data analysis was performed with the software JMP® 12 (SAS Institute Inc., Cary, NC, USA). *p* < 0.05 was considered to be significant. Although parameters had to be changed when the predicted SAR value exceeded the regulated level, we used the values obtained before changes for the evaluation.

### SNR measurements using a phantom

**Fig. 5:**
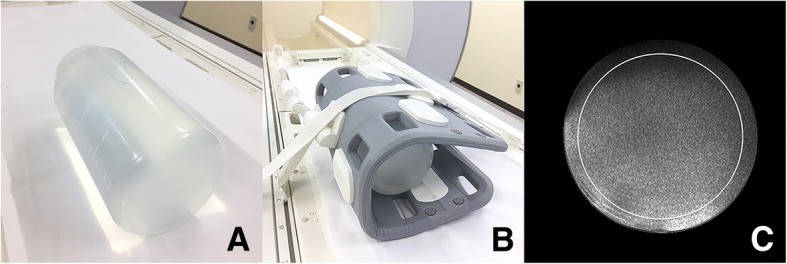
(**a**) A phantom for SNR measurements: A polypropylene cylinder filled with polyvinyl alcohol. **b** An 18-channel body array coil. **c** An SNR map and the 75% region of interest (circle)

Phantom SNRs were measured by the consecutive method to evaluate the intrinsic SNR of each protocol [11]. A polypropylene cylinder with a length of 200 mm and diameter of 120 mm filled with polyvinyl alcohol was used as a phantom (Fig. 5a). By using an identical MR scanner and the coil (Fig. 5b), scan parameters were identical to those of the patient scan. Axial PDW, T1W, and FS-T2W images were consecutively scanned 50 times each. In a series of 50 images, the signal intensity (SI) of each pixel was measured and then averaged SI and SD were also calculated. The SNR of each pixel was calculated as the SI value divided by the SD value, and SNR maps were created (Fig. 5c). In the center slice of the SNR map, the average SNR value was measured in the region of interest enclosing 75% of the area of the phantom (Fig. 5c). ImageJ software (National Institutes of Health, Bethesda, MD, USA) was used for this procedure.

## Results

The imaging times of each conventional sequence were as follows: PDW 4 min 36 s, T1W 3 min 42 s, and FS-T2W 3 min 58 s. The following imaging times were obtained with SMS: PDW 2 min 22 s, T1W 2 min 17 s, and FS-T2W 2 min 56 s (Table 1). SMS reduced the total acquisition time of the three sequences from 12 min 16 s to 7 min 35 s.

**Fig. 6:**
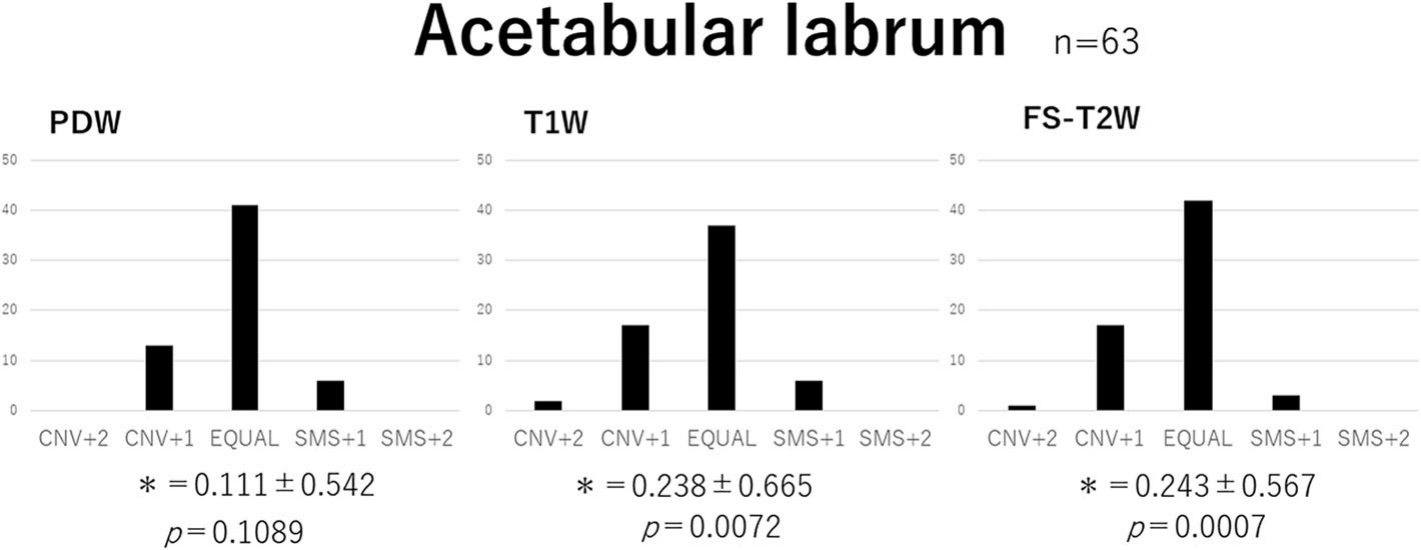
Visual image evaluation of the acetabular labrum. Bar chart of the total score obtained from three readers. Wilcoxon signed-rank test values (MEAN ± SD:*) were as follows: PDW 0.111 ± 0.542, T1W 0.238 ± 0.665, and FS-T2W 0.248 ± 0.567. No significant differences were observed in PDW, whereas T1W and FS-T2W of the labrum by conventional methods were significantly superior to those of SMS. PDW: proton density-weighted; T1W: T1-weighted; FS-T2W: fat-saturated T2-weighted; SMS: Simultaneous multi-slice; CNV: Conventional; EQUAL: equal

**Fig. 7:**
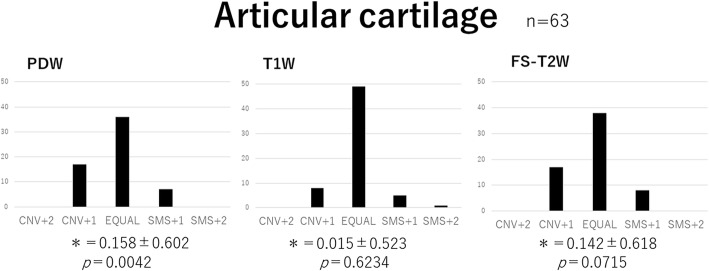
Visual image evaluation for articular cartilage. Bar chart of the total score obtained from the three readers. Wilcoxon signed-rank test values (MEAN ± SD:*) were as follows: PDW 0.158 ± 0.601, T1W 0.015 ± 0.523, and FS-T2W 0.142 ± 0.618. PDW for articular cartilage by conventional methods was significantly superior to that by SMS. No significant differences were noted in T1W and FS-T2W. PDW: proton density-weighted; T1W: T1-weighted; FS-T2W: fat-saturated T2-weighted; SMS: Simultaneous multi-slice; CNV: Conventional; EQUAL: equal

**Fig. 8:**
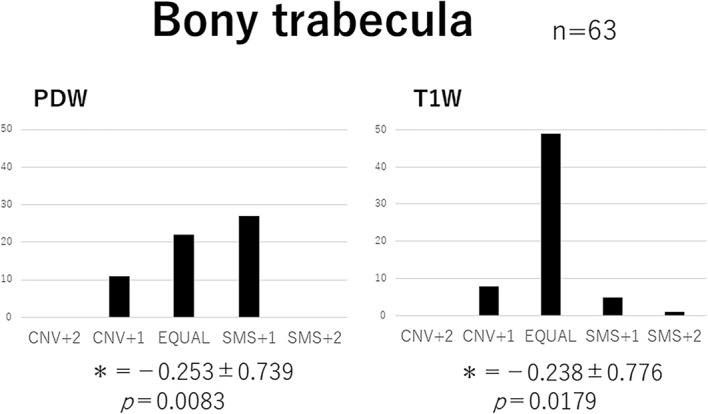
Visual image evaluation for the bony trabecula. Bar chart of the total score obtained from three readers. Wilcoxon signed-rank test values (MEAN ± SD:*) were as follows: PDW -0.253 ± 0.739 and T1W -0.238 ± 0.776. PDW and T1W for the bony trabecula by SMS were significantly superior to those by conventional methods. PDW: proton-density weighted; T1W: T1-weighted; SMS: simultaneous multi-slice; CNV: conventional; EQUAL: equal

In image evaluations, no significant differences were observed in image quality for labrum PDW and cartilage T1W and FS-T2W between conventional and SMS images. Conventional image qualities for labrum T1W (*p* = 0.0072), labrum FS-T2W (*p* = 0.0007), and cartilage PDW (*p* = 0.0042) were significantly superior to those by SMS. However, SMS image qualities for trabecula PDW (*p* = 0.0083) and T1W (*p* = 0.0179) were significantly superior to those by conventional images (Figs. 6, 7 and 8).

**Fig. 9:**
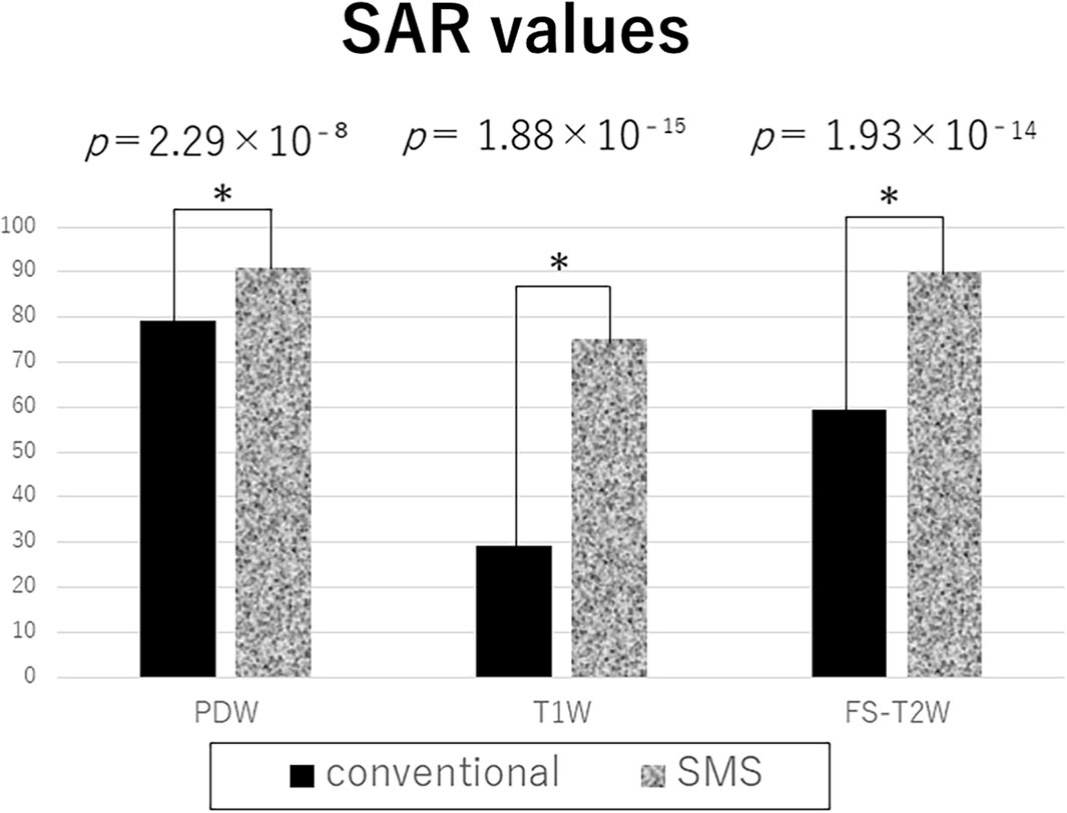
SAR values of each sequence. In all sequences, the SAR of SMS significantly increased. SAR: specific absorption rate; SMS: simultaneous multi-slice; PDW: proton density-weighted; T1W: T1-weighted; FS-T2W: fat-saturated T2-weighted

SMS images had significantly higher SAR values than conventional images (*p* < 0.001 for all sequences). The mean values of SMS sequences were within 90% of the simulated limit (Fig. 9).

Table 2 summarizes the SNR of sequences. The rates of difference (SMS/conventional) in each sequence were PDW 86%, T1W 80%, and FS-T2W 111%.

**Table 2:** Signal-to-noise ratio of each sequence

PDW		T1W		FS-T2W	
CNV	SMS	CNV	SMS	CNV	SMS
113	98	25	20	159	178

*PDW* proton-density weighted, *T1W* T1-weighted, *FS-T2W* fat-saturated T2-weighted, *CNV* Conventional, *SMS* Simultaneous multi-slice

## Discussion

The present results showed that the quality of SMS images was similar to that of conventional images and also that imaging times were reduced by 40%. None of the protocols for conventional images enabled the acquisition of all slices needed during 1TR; thus, concatenations were set to 2–3. The application of SMS resulted in fewer concatenations for all protocols and reduced imaging times.

Regarding visual assessments, conventional methods are better than SMS for bone/cartilage and soft tissues; however, no marked differences were observed on a 5-point scale basis. This result indicates the absence of any marked differences between the image qualities of SMS and conventional methods in terms of how the labrum and cartilage are shown. Regarding the bone trabecula, SMS was superior to conventional methods. Although the reason for this currently remains unclear, it is conceivable that in the evaluation of the bone trabecula, image evaluations placed more emphasis on contrast rather than shape, and image reconstruction may have reduced noise. It is advantageous for joint MRI, which requires high spatial and contrast resolution, that SMS reduced imaging times while producing similar resolution to conventional methods. Due to reductions in imaging times, it will be possible to increase the number of additions for higher resolution images and obtain more images for another section. At the hip joint, MRI is often performed on both sides of patients being treated with steroid medication [12, 13]. This reduced imaging time with SMS will be beneficial for acquiring high resolution images of both hip joints.

Image reconstruction by SMS was performed using the slice GRAPPA method, and overlapped signals were divided into each individual slice [14]. The GRAPPA kernel for SMS was calculated from the gradient echo signal acquired before the SMS scan. The arithmetic precision of this GRAPPA kernel has a prominent influence on the quality of reconstructed images. Inferior quality indicates the appearance of an artifact and decrease in SNR. In SMS, each slice was excited in order to have a different FOV shift toward the phase-encoding direction [15].With CAIPIRINHA (Controlled aliasing in parallel imaging results in higher acceleration), separation performance is significantly improved [16]. In the present study, although we set the shift amount to 1/2 FOV for slice GRAPPA factor = 2 in all protocols, the effect on a reconstructed image was expected to depend not only on these values, but also on the distance between overlapped slices, number of coils used, and slice geometry to coil configuration. Regarding the results of the phantom measurement, the SNR change was less than 20% at the maximum. Although there is a difference based on positions, no marked difference was observed from that without SMS. Slice GRAPPA may be used in conjunction with in-plane GRAPPA, but will result in a decrease in image quality due to the total acceleration factor. Although we performed a test a priori and decreased the in-plane GRAPPA factor for some protocols, we adjusted the parameters to reduce the overall imaging time.

Some points need to be emphasized for SMS: an increase in peak voltage due to a change in the radio frequency (RF) pulse shape and increase in SAR [6]. Regarding peak voltage, since it is not possible to exceed the limiting values of the hardware, methods such as prolonging the RF time and decreasing the peak voltage by shifting the peak position of each slice have been suggested [17, 18].

The SAR limitation is important for clinical use. Imaging parameters need to be changed when the SAR value exceeds its peak. Therefore, the reduced imaging time is prolonged again. Based on the measurements of SAR values for each protocol, SMS achieved high values. We need to consider patient conditions that may be more strongly affected by high SAR, such as pregnant or pediatric patients. However, the average values for all protocols were less than 100% against the limiting values, and there were only a few cases that required a change in imaging parameters during the study.

This study has several limitations. The number of subjects was small, but sufficient for a technical feasibility study. The qualitative analysis focused on the reader’s ability to evaluate intact anatomical structures. The three radiologists in our study were experienced radiologists. The addition of a relatively inexperienced reader may have increased the validity of the present study. Echo time (TE), TR, and other imaging parameters are not completely equivalent, and the resultant image contrast was greater between joint fluid and articular cartilage on the conventional scan than on the SMS scan. This contrast change may lead to the superior delineation of cartilage on PDW of a conventional scan. Furthermore, a difference exists in the magnetization transfer effect due to the simultaneous application of RF pulses with different frequencies, which may affect image contrast. In the present study, SMS imaging was performed sequentially after all conventional methods had been completed. A body motion artifact is more likely to appear due to a prolonged examination time in the magnet. In the present study, the image quality of SMS was evaluated under unfavorable conditions; however, no definite artifact was observed in the visual assessment.

The present results indicate the potential of SMS to significantly reduce imaging times and maintained equivalent image quality to conventional imaging. Images with high spatial resolution are mandatory for the evaluation of subtle morphologies in cartilage, the labrum, and trabecula. Therefore, reduced imaging times are useful for improving spatial resolution. This study approach has not been previously tested in musculoskeletal applications. Our results appear to support the replacement of conventional techniques with this imaging technique, particularly for the evaluation of musculoskeletal diseases, and applications to more advanced techniques. Therefore, the study of other joints, such as the shoulder and knee, is needed. Before actual clinical application of the new protocol, the diagnostic accuracy of the proposed image acquisition will need to be tested for chondrolabral pathology in a future study.

## Conclusions

In conclusion, the present results indicate that due to significantly shorter acquisition times and similar quality to conventional imaging, SMS may be applied to morphological evaluations of hip joint disorders without significantly increasing SAR.

## Abbreviations

CAIPIRINHA: Controlled aliasing in parallel imaging results in higher acceleration
CNV: conventional
FOV: field-of-view
FS-T2W: fat-saturated T2-weighted
GRAPPA: Generalized Auto Calibrating Partially Parallel Acquisitions
PDW: proton-density weighted
RF: radio frequency
SAR: specific absorption rate
SI: signal intensity
SMS: simultaneous multi-slice
SNR: Signal-to-noise ratio
T1W: T1-weighted
TE: echo time
TR: repetition time
TSE: turbo spin echo
